# CD209 C-Type Lectins Promote Host Invasion, Dissemination, and Infection of *Toxoplasma gondii*

**DOI:** 10.3389/fimmu.2020.00656

**Published:** 2020-04-23

**Authors:** Olivia Adhiambo Njiri, Xiaoyan Zhang, Yingmiao Zhang, Bicong Wu, Lingyu Jiang, Qiao Li, Wenqi Liu, Tie Chen

**Affiliations:** ^1^Department of Clinical Immunology, Tongji Hospital, Tongji Medical College, Huazhong University of Sciences and Technology, Wuhan, China; ^2^Department of Biological Sciences, Faculty of Science, Engineering and Technology, Chuka University, Chuka, Kenya; ^3^Division of Parasitology, Department of Pathogen Biology, School of Basic Sciences, Tongji Medical College, Huazhong University of Science and Technology, Wuhan, China

**Keywords:** *Toxoplasma gondii*, tachyzoite, C-type lectin, CD209, invasion, host dissemination

## Abstract

*Toxoplasma gondii*, the causative agent of toxoplasmosis and a major opportunistic parasite associated with AIDS, is able to invade host cells of animals and humans. Studies suggested that the ability of host invasion by the tachyzoite, the infectious form of *T. gondii*, is essential for the pathogenicity to promote its dissemination to other parts of animal hosts. However, the detailed molecular mechanisms for host invasion and dissemination of the parasites are not clear. On the other hand, viruses and bacteria are able to interact with and hijack DC-SIGN (CD209) C-type lectin on antigen presenting cells (APCs), such as dendritic cells and macrophages as the Trojan horses to promote host dissemination. In this study, we showed that invasion of *T. gondii* into host cells was enhanced by this parasite-CD209 interaction that were inhibited by ligand mimicking-oligosaccharides and the anti-CD209 antibody. Furthermore, covering the exposures of DC-SIGN by these oligosaccharides reduced parasite burden, host spreading and mortality associated with *T. gondii* infection. These results suggested that interaction of *T. gondii* to APCs expressing DC-SIGN might promote host dissemination and infection. Can the blockage of this interaction with Mannan and/or anti-CD209 antibody be developed as a prevention or treatment method for *T. gondii* infection?

## Introduction

*Toxoplasma gondii*, the causative agent of toxoplasmosis, is a protozoan, apicomplexan obligate intracellular parasite with a wide host and tissue specificity. One-third of the world's population is estimated to be infected by *T. gondii* (1–5) and more than 200 species of animals can also be infected (1). It is able to cause severe congenital infections in humans and animals and has recently attracted increased attention as an opportunistic pathogen associated with AIDS (6). Type II strains are predominant globally in immunosuppressed persons, regardless of the underlying cause of immunosuppression, site of infection or outcome. However, the genotype of *Toxoplasma* strains is strongly linked to the presumed geographical origin of infection: immunocompromised patients mostly reactivate a type II strain if acquired in Europe and an atypical strain if acquired in sub-Saharan African countries, French overseas departments or South America (7, 8). For human infection in China, studies have shown that there is a high prevalence of Type I and Chinese 1 strains in immunocompromised patients (9, 10). Toxoplasma, which can invade both phagocytic and non-phagocytic cells, requires an intracellular site for growth and replication (6, 11). *T. gondii* exists in three infectious stages: the tachyzoites (in groups or clones), the bradyzoites (in tissue cysts), and the sporozoites (in oocysts). The tachyzoites proliferate rapidly in non-intestinal epithelial cells of the definitive host and in many cells of the intermediate host (12, 13). *T. gondii* can cross the GIT (gastro-intestinal tract) in the intermediate and disseminate throughout the host in several ways particularly via intracellular migration and/or cell-dependent migration with adhesion to the host cell membrane (14). As a result, host cell invasion by the tachyzoite is critical for the pathogenicity of *Toxoplasma gondii* showing its dissemination to other parts of animal hosts (15–17). However, whether the increases of permeability by parasite invasion result in breaking the barrier during the responses to inflammatory signals, leading to the host dissemination to tissue sites e.g., the placenta, brain or eye (14, 18) remains to be determined.

Although the molecular mechanisms for host dissemination by pathogens are not clear in general, studies have shown that certain pathogens can hijack antigen presenting cells (APCs) such as dendritic cells (DCs) or macrophages by targeting the DC-SIGN (CD209) C-type lectin expressed on their surfaces to promote host dissemination (19–37). APCs express at least three immunoreceptors that belong to the calcium-dependent (C-type) lectin family: DC-specific intercellular adhesion molecule 3 grabbing non integrin (DC-SIGN, CD209), DEC-205 (CD205), and Langerin (CD207), which can be utilized by pathogens to initiate infection (20, 38–40). For example, studies indicated that several microbial pathogens like HIV and bacterial *Yersinia pestis*, the etiologic agent of plague, were able to interact with CD209s in order to hijack APCs as the Trojan horses (41) to promote host dissemination (19–37, 42–44). However, there were few such examples for studies on parasites.

Currently, toxoplasmosis chemotherapy is stage-specific and limited to the tachyzoite stage. It's difficult for treatment to completely eliminate the parasites since they usually remain within tissue cells in a quiescent state (45). New strategies that target Toxoplasma mechanisms of host–parasite interactions have been suggested for development of treatment or prevention for infection of *T. gondii* including interference with invasion and egress and/or modulation of host cell signaling and transcriptional regulation (46). Understanding the *T. gondii*-CD209 interactions would provide a possible preventive strategy for this infection.

This study therefore aimed to investigate if there is an interaction between *T. gondii* and DC-SIGN/SIGN-R1 (CD209s). If so, would this interaction show an effect on cell invasion, host dissemination, and even the infection of *T. gondii*?

## Materials and Methods

### Ethics Statement

All animal procedures were carried out in strict accordance with Standards of the People's Republic of China at protocol of (TJ-C4646). The handling of the mice and all experimental procedures were specifically approved for this study by the Medical Ethics Committee of Tongji Hospital and the Ethical Committee for Animal Experiments at the Huazhong University of Science and Technology (HUST). All procedures on mice were performed carefully to minimize suffering.

### Reagents

Mannan, the ligand antagonist of human mannose receptor; Dextran (MW 20,000, MW 40,000, MW 70,000) were purchased from Sigma-Aldrich (St. Louis, MO). Anti-mouse SIGN-R1 antibody and anti-human DC-SIGN antibody were obtained from Pharmigen (San Diego, CA, USA). For cell culture, all reagents were obtained from Hyclone PRC (gelifesciences/Hyclone), except FBS which was obtained from Zhejiang Tianhang Biotechnologies and antibiotics were obtained from Nalge NUNC, Rochester, NY). For qPCR, all reagents were purchased from Tsingke Biological Technology and Yeasen Biotechnology, PRC. For *in vivo* studies, *InVivo*MAb anti-mouse CD209b (SIGN-R1); Clone 22D1, and *InVivo*MAb polyclonal Armenian hamster IgG were purchased from BioXCell. DNA extraction reagents: Buffer GA, Buffer GB, Buffer GD, Buffer PW, Buffer TE, and Proteinase K were purchased from TIANGEN, PRC.

### Parasite Strain

*Toxoplasma gondii* (RH strain) was originally obtained from the Pathogenic Biology, Parasitology department laboratories of Tongji Medical College and used in all experiments. The RH strain was used due to its prolonged passage and stabilization of its pathogenicity in mice compared to the other non-lethal strains and its reliability in the study of host-protozoan interactions (47). The RH strain (EGFP-*T. gondii* strain) of *T. gondii* was a gift from Prof. Jilong Shen of Anhui University and was maintained in human foreskin fibroblast cells. Before being employed in the current study, the tachyzoites were peritoneally injected (5 × 10^4^ parasites per mouse) into naive mice. The second generation of tachyzoites were then recovered from the mice (ensure they are unlysed), washed and resuspended to 5 × 10^6^ parasites/ml in an invasion Endo buffer (100 ml physiological saline (NaCl) and 100 μL heparin (48, 49). Transgenic parasites used were GFP-labeled and were therefore viewed under fluorescent microscope.

### Host Cell Lines (Human and Mouse C-Type Lectin Transfectants)

CHO cells, CHO cells stably expressing mouse SIGN-R1 (CD209b) and human DC-SIGN (CD209a) cells were obtained from the Infectious Disease Department, Tongji Medical Hospital. All cells were grown at 37°C at 5% CO_2_ in Dulbecco Modified Eagle Medium (DMEM) supplemented with 10% fetal bovine serum (FBS), glutamine, and antibiotics.

CHO-mouse-SIGNR1 and CHO-human-DC-SIGN cell lines were generated by transfecting CHO cells with mouse corresponding C-type lectin cDNAs. Transfection was followed by G418 (1.5 mg ml^−1^) selection and screening for stable surface expression as originally described (50). CHO was used as a control cell line, which is an epithelial cell line that has no receptors expressed on its surface, to perform invasion assays.

#### NB

All parasites and host cells were routinely tested for *Mycoplasma* contamination by using the MycoAlert assay kit (Lonza, Basal, Switzerland).

### Mice Strains

Female C57BL/6J mice, aged 6–8 weeks, were purchased from Tongji Medical Hospital Animal Centre Laboratories (PRC). C57BL/6J-Knock-out (KO- lacking CD209) mice were bred in the Tongji Hospital Animal Centre Laboratories under pathogen-free conditions.

### Adherence and Invasion Assays

#### Harvesting Tachyzoites

In brief, tachyzoites were recovered from the peritoneum of the mice using 5ml physiological NaCl and then purified. The freshly egressed parasites were added to host cells in Endo buffer at a multiplicity of infection (MOI) of 5:1 (parasite/host cell; *N* = *10*^6^ parasites/well). Tachyzoites in the removed Endo Buffer *(*Δ*N*) were determined using a hemocytometer.

#### *In vitro* Invasion

For the invasion experiment, a multi-well (12) plate format was used to probe the invasion of parasites as described by Morisaki et al. (15) with slight modifications. Unlike receptor-mediated phagocytosis, unopsonized *Toxoplasma* cells do not bind to host cells at temperatures below 20°C, consequently, all invasion experiments were conducted at 37°C (51).

Briefly, host cells (CHO, CHO-mSIGN-R1 (CD209b), CHO-hDC-SIGN (CD209a), RAW 264.7 macrophages and peritoneal macrophages) were plated in 12-well plates and cultured overnight. The degree of cell fusion was 80%. The cells were suspended in DMEM medium with 10% FCS and double antibiotic (Nalge NUNC, Rochester, NY) at a concentration of 1 × 10^5^/ml. Twenty-four hours later, the cells were washed three times with phosphate-buffered saline (1 × PBS) and 1ml of the DMEM media added to the plates. One milliliter of parasite suspensions at a concentration of 5 × 10^6^/ml was allowed to incubate for 8–18 hours in the host cell at 37°C in the presence of 5% CO_2_. After co-culture, the cell monolayers were washed once with phosphate-buffered saline, fixed with 4% paraformaldehyde for 10–15 min, and then washed three times with 1 × PBS.

Following 8–18 h of culture, the cells were examined for lysis of infected cells and tachyzoite invasion of host cells by viewing under a fluorescence microscope (×400).

The number of internalized parasites per cell was quantified by counting the number of infected cells per view divided by the total number of cells per view. The level of internalization of parasites in the host cells was also calculated by determining the number of extracellular parasites per view.

All experiments were performed in triplicate, and each experiment was replicated thrice. The data was expressed as mean ± s.e.m.

### Receptor-Ligand Inhibition Assay

For inhibition assay, anti-mouseSIGNR1 (5 μg ml^−1^) antibody, anti-human-DC-SIGN antibody (5 μg ml^−1^) and carbohydrates including oligosaccharides [Dextran of different MW (70,000, 40,000 and 20,000) 500 μg ml^−1^] and Mannan (500μg ml^−1^) were added 20 min prior to the addition of tachyzoites (5 × 10^6^ ml^−1^). The concentrations were determined based on preliminary data and were selected based on the fact that, at these concentrations, the compounds exerted no effects on the survival of parasites and host cells as previously shown (40).

Among the oligosaccharides used, mannan (500 μg/ml) showed the highest inhibitory effect. In further experiments, Mannan was added at different concentrations (250, 500, 750, and 1,000 μg/ml) to determine the optimum concentration for the best inhibitory effect.

The rate of parasite internalization was determined as described previously and also by comparing the intensity of fluorescence-positive *T. gondii* among the different cell lines. The greater the fluorescence intensity, the more parasites are phagocytosed by host cells.

### Isolation of Mouse Peritoneal Macrophages

After euthanizing a mouse, its intact abdomen was exposed, cleaned with 70% ethanol, and opened. 5 ml of DMEM medium was injected into the intraperitoneal cavity. The mouse abdomen was gently massaged for 3 min, and then the lavage fluid collected. The suspension containing the peritoneal macrophages was seeded in 12-well plates and cultured in DMEM supplemented with 10% FCS and 1% double antibiotic at a concentration of 1 × 10^5^/ml, 1ml per well. Macrophages were then used to perform invasion assays as described previously.

### Animal Challenging for Dissemination Assay and Infection

For the animal experiment, WT C57BL/6J female mice of same genetic background were divided into 3 distinct treatment groups and each sub-group had 6 mice each. Knock-out mice used were also of the same background as the WT mice except that they lacked CD209. Western blot was performed to determine if the C-type lectin receptors had been knocked out in the mice.

#### Group 1: Treatment With Mannan I.P

Sub-group 1: Mice were injected i.p with 0.5ml physiological saline one hour pre-infection with 50 *T. gondii* (52).

Sub-group 2: Mice were injected i.p. with 250μl mannan (50mM) in 0.5ml of physiological saline one hour pre-infection with 50 *T. gondii*.

#### Group 2: Intervention With Antibody

Sub-group 1: Mice were injected i.p. with 50 μg anti–SIGN-R1 mAb (ER-TR9; BMA Biomedical) (each day from days 0 to 1) one hour prior to infection with 50 *T. gondii*.

Sub-group 2: Mice were treated with isotype control rat IgM (Serotec, Oxford, U.K.) (each day from days 0 to 1) one hour prior to infection with 50 *T. gondii* (53).

#### Group 3: Knock-Out Mice

Sub-group 1: WT mice were injected i.p with 50 *T. gondii*

Sub-group 2: KO mice were injected i.p with 50 *T. gondii*

Mice were sacrificed, and whole blood, serum, spleens and livers isolated for different procedures.

### Determination of *T. gondii* Parasitaemia by Real-Time PCR (Quantitative PCR – qRT-PCR) of DNA

In this study, the infective result was defined as *T.g* parasite burden isolated in different organs (liver and whole blood) five days post-infection from the different groups of mice.

Five (5) days post-infection, the mice were euthanized and parasite burden determined from the liver and whole blood using qRT-PCR for DNA. Briefly, DNA was extracted using the TIANamp Genomic DNA Kit according to the manufacturer's instructions. qRT-PCR was done using the BioRAD MyiQTM2 Two color real time PCR detection system (Applied Biosystems, New York, NY). Amplification of specific PCR products was detected using SYBR Green Master (Hieff qPCR SYBR GREEN master mix, Yeasen Biotechnology Co, PRC) and the following primers from TSINGKE biological technology:

TOX-s 5′CGCTGCAGGGAGGAAGACGAAAGTTG3′ and

TOX-a 5′CGCTGCAGACAGAGTGCATCTGGATT3′; and

GapDH DNA For GCAACAATCTCCACTTTGCCAC and

GapDH DNA Rev CTCACTACAGACCCATGAGGAG.

The difference in parasite load was calculated as 22DD cycle threshold using GAPDH as the housekeeping gene/control on MyiQTM2 (BIORAD, USA).

### Histopathology

Liver tissues were fixed in 4% buffered formaldehyde and embedded in paraffin wax, and 5-μm serial sections stained with H&E according to standard procedures. H&E-stained liver sections were then scored for pathology.

### Survival Studies

Mice (Wild Type C57BL/6J female mice) of the same genetic background were divided into three distinct treatment groups (those pre-treated with mannan and physiological saline; those pre-treated with anti-SIGN-R1 mAb and isotype control; and wild type and knock-out mice). Mice were injected i.p with 50-100 *T. gondii* and their survival determined in number of days.

### Statistical Analyses

Quantitative data are expressed as mean ± s.e.m. Graph Pad Prism 7.0 (Graph Pad Software, La Jolla, CA) was used for analyses. A *P*-value of 0.05 (^*^P < 0.05, ^**^P < 0.01, ^***^P < 0.0001) was considered the threshold for statistically significant differences and calculated using the Student *t-test* and one-way analysis of variance ANOVA assuming consistent Standard error mean (SEM), followed by Holm-Sidak (Tukey's multiple comparison test) post-test analysis. Survival was determined using the Gehan-Breslow-Wilcoxon Log rank (Mantel-Cox test).

## Results

### *Toxoplasma gondii* Invades Mouse Peritoneal Macrophages More Than the Macrophage Cell Line

**Figure 1:**
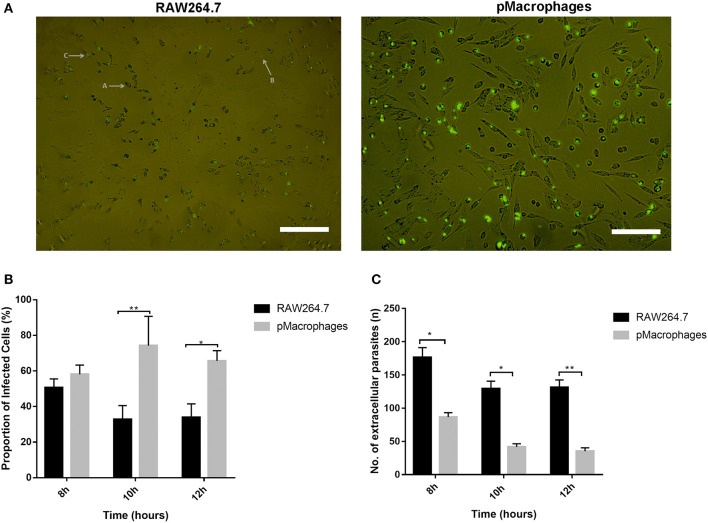
CD09 may be involved in the invasion of *T. gondii* into peritoneal macrophages. **(A)** GFP labeled intracellular parasites within adherent cells (RAW 264.7 vs. peritoneal macrophages) and extracellular parasites after 8 h of co-culture. **(B)** *T. gondii* invasion assay to determine the invasion rate of GFP-labeled *T. gondii* into peritoneal macrophages compared to macrophage cell line (RAW264.7). The cells were co-cultured with GFP-labeled *T. gondii* for 8–12 h. The number of associated parasites in different cells was quantified by counting the number of infected cells per view divided by the total number of cells per view (*P < 0.05, **P < 0.01). **(C)** The number of extracellular parasites in these sets of cells was counted (*P < 0.005, **P < 0.0001). Data shown represent one of three independent experiments with similar results.

Previous results, including some of our work, showed that APCs such as macrophages and dendritic cells (DCs) express CD209 receptors on their surface and have been shown to interact with pathogens that target CD209s for invasion (22, 24, 25, 28, 34, 36, 54–56). To determine whether *T. gondii* can interact with mouse macrophages more readily, we examined the ability of *T. gondii* to invade peritoneal macrophages that express CD209 (57, 58) compared to a macrophage cell line (RAW 264.7) that lacks the expression of CD209 receptors on its surface (59). Figure 1 and Figure S1 show invasion of GFP-labeled *T. gondii* into peritoneal macrophages and there's a cluster of parasites in some of the adherent cells, compared to that in RAW 264.7 cells. Figure S1 shows that *T. gondii* was localized both inside and outside the cell membrane. The results suggested that CD209s might play a role in interaction with *T. gondii*.

### *T. gondii* Invades CHO Cells That Express CD209s More Than CHO Cells Only

**Figure 2:**
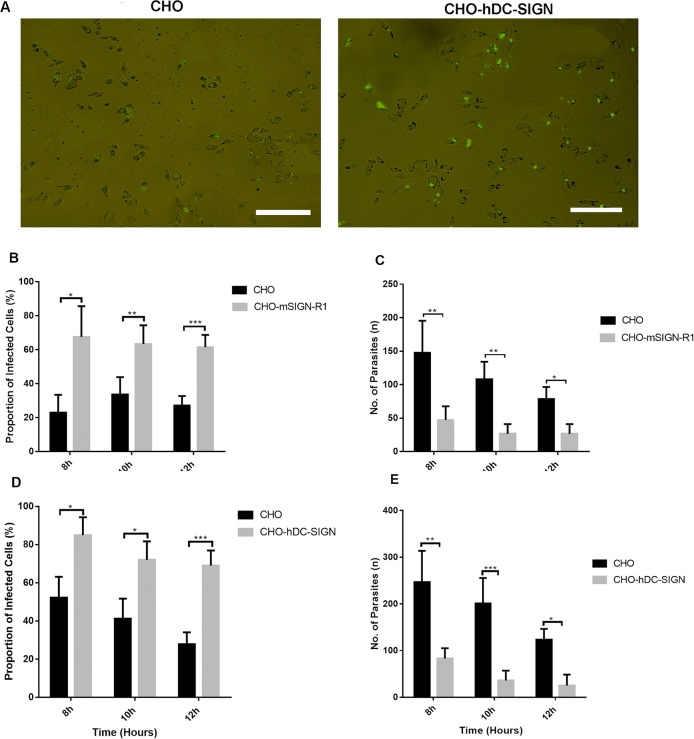
mSIGN-R1 (CD209b) and hDC-SIGN (CD209a) are receptors for *T. gondii* and play a role in invasion of *T. gondii* into host cells. **(A)** GFP labeled intracellular parasites within adherent cells (CHO vs. CHO-hDC-SIGN) and extracellular parasites after 8 h of co-culture. (**A**: Adherent cells on the wall of the plate; **B**: *T. gondii* infected cell; **C**: Extracellular parasite; **D**: A cluster of parasites infecting a cell); **(B)** Invasion assay was done to determine the invasion of *T. gondii* into CHO and other CHO transfectants expressing mSIGNR1 and **(D)** hDC-SIGN. The cells were co-cultured with GFP-labeled *T. gondii* for 8–18 h. The proportion of infected cells was determined by calculating the number of infected per view divided by the total number of cells per view (*P < 0.05, **P < 0.01, ***P < 0.001). **(C,E)** The level of internalization of *T. gondii* in CHO-mSIGNR1 **(C)** and CHO-hDC-SIGN **(E)** cells was calculated by determining the number of extracellular parasites per view (*P < 0.05). A total of 9 views were used to quantify the degree of internalization. Data are representative of three independent experiments.

To support the speculations shown in Figure 1, we tested the ability of *T. gondii* to invade CHO cells and CD209a (CHO-hDC-SIGN) and CD209b (CHO-m-SIGN-R1)-expressing CHO cells. We have been using the cell lines for many of our previous and current studies (27, 29, 30, 56, 59). Figure 2A shows that there was increased invasion and fluorescence in CHO-h-DC-SIGN than in CHO. The results also show that *T. gondii* invaded the CHO-mouse-SIGN-R1 (Figure 2B) and CHO-human-DC-SIGN (Figure 2D) more than that of CHO cells. On the other hand, the number of extracellular parasites was higher in CHO cells compared to CHO-mSIGN-R1 (Figure 2C) and CHO-hDC-SIGN (Figure 2E). Moreover, as can be seen in Figures S2 and S3, parasites clustered together to form spherical aggregates around CHO-h-DC-SIGN and fluoresced more than in CHO. This phenomenon suggests that the parasites possibly adhered to the receptor more closely since they were not visible in the cytoplasm but on the cell membrane thus the increase in the fluorescence.

In short, the data presented here showed that mSIGN-R1 and hDC-SIGN are receptors for *T. gondii* and play a role in invasion of *T. gondii* into host cells.

### Anti-human-DC-SIGN Antibody and Oligosaccharides Inhibit Receptor-Mediated Phagocytosis of *T. gondii*

**Figure 3:**
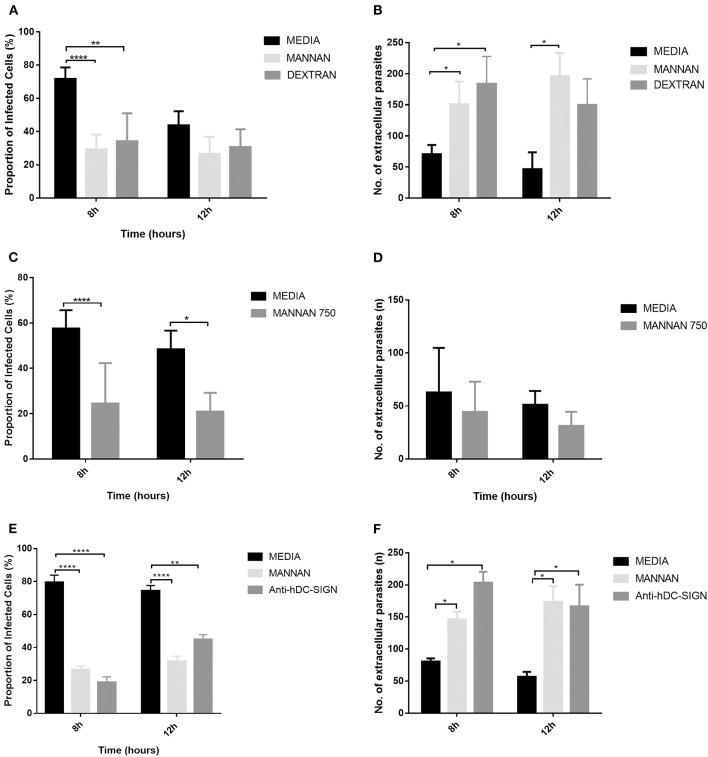
The hDCSIGN-mediated invasion of *T. gondii* was inhibited by anti-hDCSIGN antibody, Mannan, and Dextran oligosaccharides. Pre-treatment of cells with anti-hDCSIGN antibody, Mannan, and Dextran blocked invasion of the *T. gondii* into CHO-hDCSIGN. **(A)** *T. gondii* was co-cultured with CHO-hDCSIGN in the presence or absence of Mannan 500 μg ml^−1^ and Dextran of different molecular weights. The invasion rate of *T. gondii* was evaluated by determining the proportion of infected cells and the number of extracellular parasites as described in Figure 2. There was significant inhibition of invasion with Mannan and Dextran at 8 h (*P < 0.05, **P < 0.001, ****P < 0.0001, respectively). **(B)** The number of extracellular parasites was higher when inhibitors were used than in the absence of inhibitors. **(C)** Different concentrations of Mannan used to test their ability to inhibit the interaction of CHO-hDCSIGN with *T. gondii*. **(D)** There was a significant increase in the number of extracellular parasites when Mannan 250 and 500 μg ml^−1^ were used at 8 and at 10 h. **(E)** The ability of anti-hDCSIGN antibody to block this interaction was also evaluated and **(F)** the number of extracellular parasites determined. Data are representative of three independent experiments.

To validate the specific interaction of *T. gondii* and CD209, different kinds of oligosaccharides (Mannan (500 μg/ml), Dextran (500 μg/ml) and anti-human-DC-SIGN antibody were used for the receptor inhibition assay. These oligosaccharides were selected since at these concentrations they exerted no effect on the survival of parasites or host cells. Mannan is well known for its ability to block DC-SIGN-mediated interactions with HIV and other gram-negative bacteria (30, 38, 40, 60). The results show that DC-SIGN-mediated phagocytosis of *T. gondii* was inhibited by Mannan, Dextran MW390 and Dextran MW70000 (Figure 3A) and anti-human-DC-SIGN antibody (Figure 3E), signifying a specific interaction between *T. gondii* and DC-SIGN, hence promoting invasion of this parasite into the cells expressing CD209. The inhibitors had no toxic effect on the extracellular parasites but only inhibited invasion of *T. gondii* into the cells as shown in Figures 3B,D,F.

Mannan was the most commonly used inhibitor in our study since it was possibly more specific (56, 60). Mannan's inhibitory effect was therefore further tested at different concentrations to determine its efficacy even at low doses. All the concentrations of Mannan inhibited the invasion of *T. gondii* to CHO-hDC-SIGN and did not affect the parasite survival. The inhibitory effect also increased with increasing concentration (Figure 3C). However, in Figure 3D, there was a reduction in the number of extracellular parasites when Mannan 750 μg/ml was used as opposed to what was observed when Mannan 500 μg/ml was used showing a possible toxic effect of a higher concentration of Mannan on extracellular parasites. Similar results were seen when Mannan 1,000 μg/ml was used (results not shown).

While Mannan and anti-human-DC-SIGN were used, most of the parasites appeared singly and thus did not fluoresce as much as when inhibitors were not used. In this case (CHO-hDC-SIGN), the parasites appeared in clusters and thus fluoresced more. This implies that, at optimum concentration, the inhibitors prevented both the binding of the tachyzoites to the cells and their subsequent invasion but did not affect their survival.

### Anti-mouse-SIGNR1 Antibody, Mannan, and CD209-Knock-Out Mice Inhibit *T. gondii* Dissemination

In addition, to investigate the contribution of CD209 to dissemination, we blocked CD209 expressing cells *in vivo* by pre-treating mice with antibodies specific to CD209 and Mannan before infecting the mice with *T. gondii* i.p. We also used CD209-knock-out (KO) mice (CD209^−/−^) and compared dissemination of *T. gondii* with that of wild type mice. The mice were sacrificed 5 days post-infection and qRT-PCR of *T. gondii* DNA used to assess parasitaemia in their blood and liver. Our results show that there was decreased parasite burden in the liver and blood of knock-out, antibody- and mannan- pre-treated mice compared to the liver and blood of wild type, isotype- and physiological saline- pre-treated mice respectively (**Figures 5A–C**). This implies that the receptor blocking *in vivo* also prevented invasion and dissemination of *T. gondii* in blood and in liver but other factors could be at play within the liver tissues.

### The CD209s Were Involved in the *Toxoplasma* Induced Liver Damage, Infection, and Mortality, Which Was Reduced by Pre-treating the Mice With Mannan and/or Anti-CD209 Antibody

**Figure 4:**
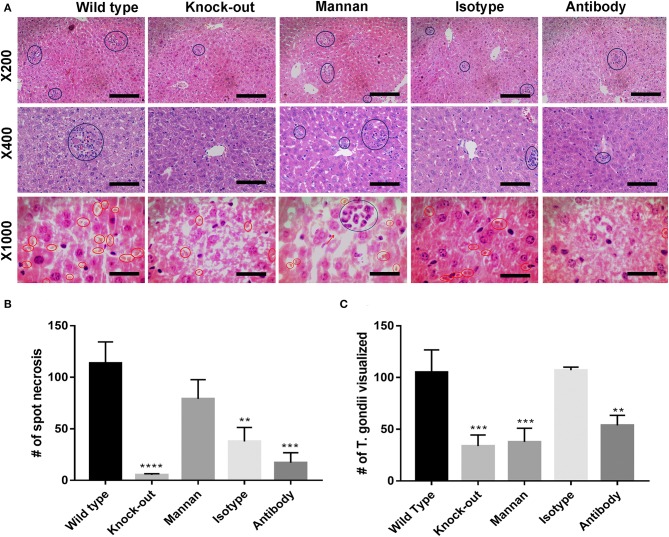
*T. gondii* was able to be disseminated to the livers, which was reduced in CD209-knock-out mice, and by addition of mannan oligosaccharides and anti-mSIGNR1 antibody. *T. gondii* dissemination ability to liver tissues was examined by determining hepatic pathological changes in mice five days post-infection with *T. gondii*. **(A)** Representative images of H&E stained liver sections from different groups of mice (*n* = 6 mice/group). Blue circles indicate spot necrosis and red circles indicate *T. gondii* tachyzoites visualized within tissue sections. First level shows MG = X200, second level MG = X400, and third level shows MG = X1000. KO mice and antibody pre-treated mice had less inflammatory foci indicating they had less parasites disseminating within them. **(B)** The number of spot necrosis was counted. Mean + s.e.m results are graphed (**P < 0.01, ***p < 0.005, ****p < 0.0001). **(C)** *T. gondii* visualized within liver sections was also counted and the Mean + s.e.m results are graphed (***p < 0.005, ****p < 0.0001). A total of 9 H&E sections per group of mice were used to quantify the number of inflammatory foci and of tachyzoites visualized. Data are representative of three independent experiments with similar results.

To determine pathological changes, infected mice were sacrificed 5-days post-infection and liver sections examined for morphology using H&E staining. There was more damage in wild type liver tissues that showed more inflammation, lymphocyte infiltration, and spot necrosis than the KO mice and the antibody-pretreated mice (Figure 4). Spot necrosis was distributed throughout the liver tissues at different degrees in different groups of mice and this damage coincided with hydratic degeneration indicated with pale cytoplasm around the region with lymphocyte infiltration as shown in Figures 4A,B. Liver damage therefore occurred in all groups of mice but at different levels.

The number of *T. gondii* visualized in the tissue sections was also counted as a measure of parasite burden. The results (Figure 4C) show that the highest number of tachyzoites in liver sections was observed in wild type and isotype-pretreated mice which coincided well with the number of spot necrosis (Figure 4B). However, it was interesting to note that, Mannan had a higher number of spot necrosis, more hydratic degeneration which was an indication of oedema, but a lower number of tachyzoites in the liver sections.

**Figure 5:**
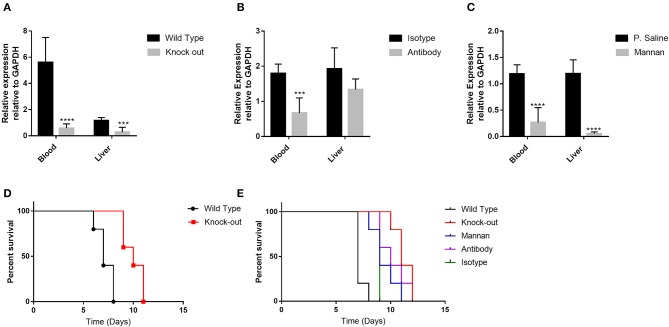
The infectivity of *T. gondii* was reduced by pre-treatment of mice with anti-mSIGN-R1 antibody and certain oligosaccharides. **(A)** *T. gondii* dissemination ability to liver tissues and blood was examined in wild type and knock-out mice by determining parasite burden in the blood and liver sections using qPCR of DNA. **(B)** Dissemination of *T. gondii* in the liver and blood of mice pre-treated with isotype antibody and anti-mSIGNR1 antibody was also examined as described above and **(C)** Dissemination of *T. gondii* in the liver and blood of mice pre-treated with Mannan and physiological saline was also determined in a similar manner. Reduction in *T. gondii* dissemination to blood and liver was observed in the knock out mice, and mice pre-treated with mannan and anti-mSIGN-R1 antibody. **(D)** Survival rates of wild type and knock-out mice post-infection with *T. gondii*. **(E)** Survival rates of different groups of mice after infection with *T. gondii*. For each group, six mice were infected with 50–100 *T. gondii* and observed until they all died (12 days post-infection). Log rank was performed. The knock-out mice had the highest survival percentage post infection compared to the other groups of mice (*P < 0.05, ***P < 0.005, ****P < 0.0001). The data shown are obtained from three independent experiments with similar results.

To elucidate the survival of infected mice, 50–100 *Toxoplasma gondii* was inoculated into the mice i.p and the survival time determined in terms of number of days. Among all the sub-groups of mice, the KO mice had the highest survival rate compared to the wild type mice (*P* = 0.0181) and also compared to the pre-treated mice using mannan and/or antibody (*P* = 0.0031) (Figures 5D,E). They all died within 8 days but the KO survived up to 12 days post-infection. These results suggest that despite the virulent nature of RH strain *T. gondii*, lack of CD209 receptor provided some form of protection to the KO mice.

## Discussion

*T. gondii* has the ability to cross the GIT in the intermediate host and spread in various ways specifically via intracellular migration and/or cell-dependent migration with adhesion to the host cell membrane by employing a Trojan horse following increased permeability of the barrier in response to inflammatory signals (14, 41, 61–65). Studies have shown that pathogens such as viral HIV and bacterial *Y. pestis* were able to bind CD209 to hijack APCs as the Trojan horses for host dissemination (19, 42, 44). In this study, our results suggested that *T. gondii* may use the similar mechanisms for its host dissemination.

*T. gondii*, which has oligomannose on its surface membrane (66) may be recognized and bound by CD209. A mAb specific to the CD209 and an oligosaccharide (Mannan) which specifically binds mannose-related receptors and ligands, inhibited the invasion of *T. gondii* into the CHO-hDC-SIGN (CD209a) and CHO-mSIGN-R1 (CD209b) *in vitro* (Figure 3) and the effect was not reversed when the inhibitors were washed off prior to addition of the parasites (Figures 3A,C,E). Although there was decreased invasion when a higher mannan concentration (750 μg/ml) was used (Figure 3C), fewer extracellular parasites (Figure 3D) were observed as opposed to what was expected i.e., similar results as when Mannan 500 μg/ml was used. We have speculated that the inhibitory effect of Mannan was retained, thus a lower percentage of intracellular parasites but due to the higher concentration, it is possible that some extracellular parasites were killed and led to a reduction in their number. It is therefore possible that 500 μg/ml is still the optimum concentration for the best inhibitory effect that will not affect the survival of the parasites. However, further investigations need to be done to validate this phenomenon. Although *T. gondii* infection was facilitated via the CD209 receptor, it was not the only target for the parasite since it can also bind to other phagocytic and non-phagocytic cells in the host (14, 15, 17). This suggested that CD209 mainly enhanced infection of *T. gondii* and was a preferred target by the parasite for infection and dissemination.

In mice infected with *T. gondii*, receptor blockade prior to peritoneal infection decreased parasite burden, dissemination and pathogenicity. However, this raises the question how this is possible since studies have shown that *T. gondii* infection relies primarily on active invasion of host cells (15) rather than phagocytic uptake through receptors like CD209. Invasion often occurs as a result of active gliding motility when motile *T. gondii* contact the host cell with their apical end. Invasion is thus considerably faster than phagocytosis, however, even after invasion, some parasites are still able to egress the cells and eventually die (48) probably making phagocytosis more efficient in sustaining *T. gondii* within the cells after invasion.

During host cell invasion following phagocytosis, *T. gondii* attaches to the host cell with its apical end. A variety of MIC proteins participate in this cell attachment (67, 68) and our study has shown that CD209 could be the possible corresponding host cell receptors. These interactions facilitate repeated rounds of attachment and release that would be expected to support gliding motility (67). The ability of *T. gondii* to invade and not egress DCs and macrophages (during phagocytosis) is therefore an important adaptation for dissemination to tissues within the body.

Similar results were reported in a study involving *T. musculi* and *T. cruzi* whose results showed enhanced survival in macrophages, whereas receptor blockade decreased parasite growth (69). A number of studies using different antibodies and different strains of knock-out mice have yielded a wide array of contradicting results (11, 70–74). In our study knock-out mice survived longer than the other groups of mice post-infection followed by Mannan and antibody pre-treated mice. Pathological damage of the liver was more in wild type mice and isotype pre-treated mice compared to the other groups of mice (knock-out, and antibody-pretreated mice) which had less pathological damage. Therefore the lack of CD209 or its blockade protected the liver from severe pathological effects of *T. gondii* and provided optimal resistance to the infection. The presence of CD209 enhanced *T. gondii* invasion, dissemination and thus infection. This possibly leads to an increase in the rate of parasite proliferation, decreased immunity and ultimately increased mortality as observed in the wild type and isotype-pre-treated mice. Further studies may reveal whether increased immune responses would have been involved in the detrimental effect on the host tissues.

The wild type mice had lower parasite burden in the liver than blood (Figure 4A) compared to other groups of mice which had almost the same parasite burden in both tissues. Interestingly, in the antibody-pre-treated mice, the parasite load was higher in liver tissues as compared to blood (Figure 4B). The higher levels in the liver could be due to other factors at play within the liver that could have been triggered by pre-treatment of mice using antibody prior to infection with *T. gondii* (75) causing them to remain localized in the liver instead of migrating to other tissues like MLNs and portal lymphatic vessels. Generally, the parasite burden was still lower in the antibody-pretreated group compared to the isotype-pre-treated group (Figure 4B) indicating a possible form of protection against the parasite. In case of wild type mice, the parasitaemia could have been higher in the liver but since the liver DCs are still migratory in nature due to the presence of CD209 (75), they could have migrated to other organs thus not detected in the liver resulting to lower parasitaemia in liver tissues as compared to blood. On the other hand, Mannan pre-treated mice had a higher number of spot necrosis but lower tachyzoites visualized within the tissues (Figure 4B). It is not clear why such a response was observed but probably due to other immune factors at play within the liver which triggered an increase in lymphocyte infiltration and hydratic degeneration.

CD209 plays diverse roles in the innate immune system by inducing various immune responses to pathogens (32, 37, 39). Several pathogens exploit DC-SIGN to suppress an efficient immune response (37). However, in the KO mice, the lack of CD209 protected the mice from *T. gondii* infection. This could possibly be because *T. gondii* is highly specific to DC-SIGN and also due to the lethal nature of the parasite. On the contrary, previous studies using *Yersinia pseudotuberculosis* showed that partial inhibition of CD209 between the interaction of hDCS and *Y. pseudotuberculosis* was achieved only when the triple combination of inhibitors (antireceptor antibodies, mannan oligosaccharide, and purified LPS core) was employed. In this study, even the use of a single inhibitor provided partial protection against *Toxoplasma* infection as was also observed in *Trypanosoma musculi* infection (69).

We have shown that the possible host receptors involved in *T. gondii* infection, but the specific parasite molecules that bind to DC-SIGN were not identified. It is well-documented that the surfaces of most parasites are highly glycosylated (11, 27, 29, 76). Further investigations need to be done to determine corresponding parasite ligands, as we did in identification of the carbohydrate LPS core from many Gram-negative bacteria (29, 30, 38, 40, 56, 59); as well as immune responses elicited by the *T. gondii*. Moreover, HIV uses gp120–DCSIGN interaction to initiate capture by DCs and transmission to CD4+ T cells (19). Therefore, blockage of DC-SIGN-mediated transmission of HIV (31, 77, 78) and *Y. pestis* (29, 30, 40) are recognized as valid therapeutic strategies to fight HIV infection and plague.

DC-SIGN mediates HIV-1 internalization and studies on milk and serum proteins were done to test their ability to block DC-mediated HIV-1 transmission. The best inhibitor was bovine lactoferrin (bLF) which binds strongly to DC-SIGN than human lactoferin, thus preventing virus capture and further transmission. This highlights the usefulness of bLF as a microbicide drug to prevent HIV-1 transmission (77).

In summary, our study has shown that *T. gondii* is able to disseminate to other parts of mammalian organs and cause systemic infections by potentially utilizing the CD209s. It has thus disclosed a possible treatment strategy or prevention of *T. gondii* infection by blocking its interaction with C-type lectins using certain oligosaccharides like mannan, and specific blocking antibodies which reduced parasite burden in the mice, protected the liver from severe pathological damage and reduced mortality of the infected mice.

## Data Availability Statement

All datasets generated for this study are included in the article/Supplementary Material.

## Ethics Statement

The animal study was reviewed and approved by Medical Ethics Committee of Tongji Hospital and the Ethical Committee for Animal Experiments at the Huazhong University of Science and Technology.

## Author's Note

*Toxoplasma gondii* is a parasitic pathogen that causes blindness, mental problems, and mother-child infection especially in those infected with HIV/AIDS. This is because this parasite can spread to almost any part of the body particularly the eyes, brain, placenta, and cause infection in animals and humans. However, how the parasite spread at the cellular and molecular level is still unclear. Our body uses various defense mechanisms or weapons like macrophages of host cells to protect itself against invading pathogens. The initial goal of the host cells is to eat and kill the pathogens. At the same time, the macrophages can move pathogens to different parts of the body, seeking for additional help to destroy the pathogens. However, this study has shown that *T. gondii* may interact and hijack the macrophages as Trojan horses to spread to different parts of the body. This study could therefore open avenues for developing new preventive and treatment regimens by blocking the interaction between the host cells and *T. gondii*.

## Author Contributions

TC, WL, and ON contributed to conceptualization and design of the study. ON, XZ, YZ, and BW performed the assays. ON, LJ, and QL visualized the results and performed interpretation of data and statistical analysis. ON and QL wrote the first draft of the manuscript. All authors contributed to manuscript revision, read, and approved the submitted version.

## Conflict of Interest

The authors declare that the research was conducted in the absence of any commercial or financial relationships that could be construed as a potential conflict of interest.

## References

[1] Hill D Dubey JP. *Toxoplasma gondii*: transmission, diagnosis and prevention. Clin Microbiol Infect. (2002) 8:634–40. 10.1046/j.1469-0691.2002.00485.x 12390281

[2] Zhou P Zhaoguo C Hai L Li H Zheng S He RQ. *Toxoplasma gondii* infection in humans in China. Paras Vectors. (2011) 4:1–9. 10.1186/1756-3305-4-165 21864327 PMC3174123

[3] Chaichan P Mercier A Galal L Mahittikorn A Ariey F Morand S. (2017). Geographical distribution of *Toxoplasma gondii* genotypes in asia: a link with neighboring continents. Infect Genetics Evol. 53:227–38. 10.1016/j.meegid.2017.06.002 28583867

[4] Pan M Lyu C Zhao J Shen B. Sixty years (1957–2017) of research on toxoplasmosis in China—an overview. Front Microbiol. (2017) 8:1–16. 10.3389/fmicb.2017.01825 28993763 PMC5622193

[5] Galal L Ajzenberg D Hamidović A Durieux M-F Dardé M-L Mercier A. Toxoplasma and Africa: one parasite, two opposite population structures. Trends Parasitol. (2018) 34:140–54. 10.1016/j.pt.2017.10.010 29174610

[6] Uttah E Ogban E Okonofua C Toxoplasmosis: a global infection, so widespread, so neglected. Int J Sci Res Pub. (2013) 3:2250–3153.

[7] Ajzenberg D Banuls AL Su C Dumetre A Demar M Carme B. Genetic diversity, clonality and sexuality in *Toxoplasma gondii*. Int J Parasitol. (2004) 34:1185–96. 10.1016/j.ijpara.2004.06.007 15380690

[8] Hosseini SA Amouei A Sharif M Sarvi S Galal L Javidnia J (2018). Human toxoplasmosis: a systematic review for genetic diversity of *Toxoplasma gondii* in clinical samples. Epidemiol Infect. (2018) 147:1–9. 10.1017/S0950268818002947 PMC6518561 30394261

[9] Khan AC Su M German GA Storch DB Clifford L Sibley D. Genotyping of *Toxoplasma gondii* strains from immunocompromised patients reveals high prevalence of type I strains. J Clin Microbiol. (2005) 43:5881–87. 10.1128/JCM.43.12.5881-5887.2005 16333071 PMC1317192

[10] Zhou P Zhang H Lin R-Q Zhang D-L Song H-Q Chunlei SU. Genetic characterization of *Toxoplasma gondii* isolates from China. Parasitol Int. (2009) 58:193–5. 10.1016/j.parint.2009.01.006 19567233

[11] Han SJ Visualization of Immune Cells During Toxoplasma gondii Infection. Dissertation. der Freien Universitat Berlin (2012). p. 1–46.

[12] Dubey JP. The history of *Toxoplasma gondii* - the first 100 years. J Eukar Microbiol. (2008) 55:467–75. 10.1111/j.1550-7408.2008.00345.x 19120791

[13] McAuley JB. Congenital toxoplasmosis. J Pediatr Infect Dis Soc. (2014) 3(suppl_1):S30–5. 10.1093/jpids/piu077 25232475 PMC4164182

[14] Randall LM Hunter CA. Parasite dissemination and the pathogenesis of toxoplasmosis. Euro J Microbiol Immunol. (2011) 1:3–9. 10.1556/EuJMI.1.2011.1.3 24466431 PMC3894809

[15] Morisaki JH Heuser JE Sibley LD. Invasion of *Toxoplasma gondii* occurs by active penetration of the host cell. J Cell Sci. (1995) 108:2457–64. 10.3891/acta.chem.scand.16-2357 7673360

[16] Sibley LD. *Toxoplasma gondii*: perfecting an intracellular life style. Traffic. (2003) 4:581–6. 10.1034/j.1600-0854.2003.00117.x 12911812

[17] Sweeney KR Morrissette NS LaChapelle S Blader IJ. Host cell invasion by *Toxoplasma gondii* is temporally regulated by the host microtubule cytoskeleton. Eukaryot Cell. (2010) 9:1680–9. 10.1128/EC.00079-10 20435700 PMC2976295

[18] Mordue DG Monroy F La Regina M Dinarello CA Sibley LD. Acute toxoplasmosis leads to lethal overproduction of Th1 cytokines. J Immunol. (2001) 167:4574–84. 10.4049/jimmunol.167.8.4574 11591786

[19] Geijtenbeek TBH Kwon DS Torensma R Van Vliet SJ Van Duijnhoven GCF Middel J. DC-SIGN, a dendritic cell – specific HIV-1-binding protein that enhances trans -infection of T cells. Cell. (2000) 100:587–97. 10.1016/s0092-8674(00)80694-7 10721995

[20] Colmenares M Puig-Kröger A Pello OM Corbí AL Rivas L. Dendritic cell (DC)-specific intercellular adhesion molecule 3 (ICAM-3)-grabbing nonintegrin (DC-SIGN, CD209), a C-type surface lectin in human DCs, is a receptor for leishmania amastigotes. J Biol Chem. (2002) 277:36766–9. 10.1074/jbc.M205270200 12122001

[21] Appelmelk BJ van Die I van Vliet SJ Vandenbroucke-Grauls CMJE Geijtenbeek TBH van Kooyk Y. Cutting edge: carbohydrate profiling identifies new pathogens that interact with dendritic cell-specific ICAM-3-grabbing nonintegrin on dendritic cells. J Immunol. (2003) 170:1635–9. 10.4049/jimmunol.170.4.1635 12574325

[22] Cambi A Gijzen K de Vries IJM Torensma R Joosten B Adema GJ. The C-type lectin DC-SIGN (CD209) is an antigen-uptake receptor for *Candida albicans* on dendritic cells. Euro J Immunol. (2003) 33:532–8. 10.1002/immu.200310029 12645952

[23] Geijtenbeek TB Van Vliet SJ Koppel EA Sanchez-Hernandez M Vandenbroucke-Grauls CM Appelmelk B. Mycobacteria target DC-SIGN to suppress dendritic cell function. J Exp Med. (2003) 197:7–17. 10.1084/jem.20021229 12515809 PMC2193797

[24] Van Die I van Vliet SJ Nyame AK Cummings RD Bank CMC Appelmelk B. The dendritic cell-specific C-type lectin DC-SIGN is a receptor for schistosoma mansoni egg antigens and recognizes the glycan antigen Lewis X. Glycobiology. (2003) 13:471–8. 10.1093/glycob/cwg052 12626400

[25] Ludwig IS Lekkerkerker AN Depla E Bosman F Musters RJ Depraetere S Hepatitis C virus targets DC-SIGN and L-SIGN to escape lysosomal degradation. Edited by Paul Klenerman. J Virol. (2004) 78:8322–32. 10.1128/JVI.78.15.8322-8332.2004 15254204 PMC446128

[26] Caparros E Serrano D Puig-Kroger A Riol L Lasala F Martinez I. Role of the C-type lectins DC-SIGN and L-SIGN in leishmania interaction with host phagocytes. Immunobiology. (2005) 210:185–93. 10.1016/j.imbio.2005.05.013 16164025 PMC7114652

[27] Klena J Zhang P Olivier S Hull S Chen T. The core lipopolysaccharide of *Escherichia coli* is a ligand for the dendritic-cell-specific intercellular adhesion molecule nonintegrin CD209 receptor. J Bacteriol. (2005) 187:1710–5. 10.1128/JB.187.5.1710-1715.2005 15716442 PMC1064026

[28] Tailleux L Pham-Thi N Bergeron-Lafaurie A Herrmann J-L Charles P Schwartz O. (2005). DC-SIGN induction in alveolar macrophages defines privileged target host cells for mycobacteria in patients with tuberculosis. PLoS Med. 2:e381. 10.1371/journal.pmed.0020381 16279841 PMC1283365

[29] Zhang P Snyder S Feng P Azadi P Zhang S Bulgheresi S. Role of N-acetylglucosamine within core lipopolysaccharide of several species of gram-negative bacteria in targeting the DC-SIGN (CD209). J Immunol. (2006) 177:4002–11. 10.4049/jimmunol.177.6.4002 16951363

[30] Zhang P Skurnik M Zhang S-S Schwartz O Kalyanasundaram R Bulgheresi S. Human dendritic cell-specific intercellular adhesion molecule-grabbing nonintegrin (CD209) is a receptor for yersinia pestis that promotes phagocytosis by dendritic cells. Infect Immun. (2008) 76:2070–9. 10.1128/IAI.01246-07 18285492 PMC2346686

[31] Gringhuis SI den Dunnen J Litjens M van der Vlist M Geijtenbeek TB. Carbohydrate-specific signaling through the DC-SIGN signalosome tailors immunity to mycobacterium tuberculosis, HIV-1 and *Helicobacter pylori*. Nat Immunol. (2009) 10:1081–88. 10.1038/ni.1778 19718030

[32] Kerrigan AM Brown GD. C-type lectins and phagocytosis. Immunobiology. (2009) 214:562–75. 10.1016/j.imbio.2008.11.003 19261355 PMC2702671

[33] Günther PS Mikeler E Hamprecht K Schneider-Schaulies J Jahn G Dennehy KM. CD209/DC-SIGN mediates efficient infection of monocyte-derived dendritic cells by clinical adenovirus 2C isolates in the presence of bovine lactoferrin. J Gen Virol. (2011) 92(Pt 8):1754–9. 10.1099/vir.0.030965-0 21562123

[34] Lozach P-Y Kuhbacher A Meier R Mancini R Bitto D Bouloy M. DC-SIGN as a receptor for phleboviruses. Cell Host Microbe. (2011) 10:75–88. 10.1016/j.chom.2011.06.007 21767814

[35] Sanecka A Frickel E-M. Use and abuse of dendritic cells by *Toxoplasma gondii*. Virulence. (2012) 3:678–89. 10.4161/viru.22833 23221473 PMC3545950

[36] Goncalves A-R Moraz M-L Pasquato A Helenius A Lozach P-Y Kunz S. Role of DC-SIGN in lassa virus entry into human dendritic cells. J Virol. (2013) 87:11504–15. 10.1128/JVI.01893-13 23966408 PMC3807329

[37] Geijtenbeek TBH Kooyk YV. Pathogens target DC-SIGN to influence their fate: DC-SIGN functions as a pathogen receptor with broad specificity. Apmis. (2003) 111:698–714. 10.1034/j.1600-0463.2003.11107803.x 12974773

[38] Zhang SS Park CG Zhang P Bartra SS Plano GV Klena JD. Plasminogen activator pla of yersinia pestis utilizes murine DEC-205 (CD205) as a receptor to promote dissemination. J Biol Chem. (2008) 283:31511–21. 10.1074/jbc.M804646200 18650418 PMC2581554

[39] van den Berg LM Gringhuis SI Geijtenbeek TBH. An evolutionary perspective on C-type lectins in infection and immunity. Annals NY Acad Sci. (2012) 1253:149–58. 10.1111/j.1749-6632.2011.06392.x 22288724

[40] Yang K Park CG Cheong C Bulgheresi S Zhang S Zhang P. Host langerin (CD207) is a receptor for yersinia pestis phagocytosis and promotes dissemination. Immunol Cell Biol. (2015) 93:815–24. 10.1038/icb.2015.46 25829141 PMC4612776

[41] Lencer WI Neutra MR. Salmonella pathogenesis: the Trojan horse or the New York shuttle? Gastroenterology. (2000) 118:803–5. 10.1016/s0016-5085(00)70153-3 10734034

[42] McDonald D Wu L Bohks SM KewalRamani VN Unutmaz D Hope TJ. Recruitment of HIV and its receptors to dendritic cell-T cell junctions. Science. (2003) 300:1295–7. 10.1126/science.1084238 12730499

[43] Cunningham AL Harman AN Donaghy H. DC-SIGN ‘AIDS' HIV immune evasion and infection. Nat Immunol. (2007) 8:556–8. 10.1038/ni0607-556 17514207

[44] Yang K He Y Park CG Kang YS Zhang P Han Y. Yersinia pestis interacts with SIGNR1 (CD209b) for promoting host dissemination and infection. Front Immunol. (2019) 10:96. 10.3389/fimmu.2019.00096 30915064 PMC6422942

[45] CDC-Global Health-Division of Parasitic Diseases and Malaria CDC - Toxoplasmosis - Prevention & Control. (2013). Available online at: https://www.cdc.gov/parasites/toxoplasmosis/prevent.html (accessed January 20, 2020).

[46] McFarland MM Zach SJ Wang X Potluri LP Neville AJ Vennerstrom JL. Review of experimental compounds demonstrating anti-toxoplasma activity. Antimicrob Agents Chemother. (2016) 60:7017–34. 10.1128/AAC.01176-16 27600037 PMC5118980

[47] Asgari Q Keshavarz H Shojaee S Hossein M Mehdi M Ramin M. *In vitro* and *in vivo* potential of RH strain of‘ *Toxoplasma gondii*. (Type I) in tissue cyst forming. Iran J Parasitol. (2013) 8:367–75. Available online at: https://www.ncbi.nlm.nih.gov/pubmed/24454428 24454428 PMC3887236

[48] Kafsack BF Carruthers VB Pineda FJ. Kinetic modeling of *Toxoplasma gondii* invasion. J Theor Biol. (2007) 249:817–25. 10.1016/j.jtbi.2007.09.008 17942124 PMC2692516

[49] Khan A Grigg ME. *Toxoplasma gondii*: laboratory maintenance and growth. In: Current Protocols in Microbiology. Hoboken, NJ: Wiley Publishing Company (2017). 10.1002/cpmc.26 PMC5537724 28166387

[50] Kang YS Yamazaki S Iyoda T Pack M Bruening SA Kim JY. SIGN-R1, a novel C-type lectin expressed by marginal zone macrophages in spleen, mediates uptake of the polysaccharide dextran. Int Immunol. (2003) 15:177–86. 10.1093/intimm/dxg019 12578847

[51] Walker DM Oghumu S Gupta G McGwire BS Drew ME Satoskar AR. Mechanisms of cellular invasion by intracellular parasites. Cell Mol Life Sci. (2013) 71:1245–63. 10.1007/s00018-013-1491-1 24221133 PMC4107162

[52] Turner PV Brabb T Pekow C Vasbinder MA. Administration of substances to laboratory animals: routes of administration and factors to consider. J Am Assoc Lab Anim Sci. (2011) 50:600–613. 10.1016/j.jad.2013.05.099 22330705 PMC3189662

[53] Saunders SP Barlow JL Walsh CM Bellsoi A Smith P McKenzie AN. C-type lectin SIGN-R1 has a role in experimental colitis and responsiveness to lipopolysaccharide. J Immunol. (2010) 184:2627–37. 10.4049/jimmunol.0901970 20130211

[54] Serrano-Gómez D Domínguez-Soto A Ancochea J Jimenez-Heffernan JA Leal JA Corbí AL. Dendritic cell-specific intercellular adhesion molecule 3-grabbing nonintegrin mediates binding and internalization of *Aspergillus fumigatus* conidia by dendritic cells and macrophages. J Immunol. (2004) 173:5635–43. 10.4049/jimmunol.173.9.5635 15494514

[55] Cassado AA Lima MRD Bortoluci KR Revisiting mouse peritoneal macrophages: heterogeneity, development, and function. Front Immunol. (2015) 6:1–9. 10.3389/fimmu,.2015.00225 26042120 PMC4437037

[56] He YX Ye CL Zhang P Li Q Park CG Yang K. Yersinia pseudotuberculosis exploits CD209 receptors for promoting host dissemination and infection. Infect Immun. (2019) 87:18. 10.1128/IAI.00654-18 30348825 PMC6300620

[57] Zhang X Goncalves R Mosser DM The isolation and characterization of murine macrophages. Curr Protoc Immunol. (2008) 83:14.1.1–14.1.14. 10.1002/0471142735.im1401s83 PMC2834554 19016445

[58] Liao C-T The Study of Macrophage Heterogeneity in the Peritoneal Cavity. (2015). Available online at: https://pdfs.semanticscholar.org/3a80/3e46906090f294ee540a7e83049e81cbd6dd.pdf (accessed January 20, 2020).

[59] Ye C Li Q Li X Park CG He Y Zhang Y. *Salmonella enterica* serovar typhimurium interacts with CD209 receptors to promote host dissemination and infection. Infect Immun. (2019) 87:19. 10.1128/IAI.00100-19 31085704 PMC6652768

[60] Zhang P Schwartz O Pantelic M Li G Cinzia K Milan N. DC-SIGN (CD209) recognition of neisseria gonorrhoeae is circumvented by lipooligosaccharide variation. J Leukoc Biol. (2006) 79:731–8. 10.1189/jlb.0405184 16461738

[61] Moudy R Manning TJ Beckers CJ. The loss of cytoplasmic potassium upon host cell breakdown triggers egress of *Toxoplasma gondii*. J Biol Chem. (2001) 276:41492–501. 10.1074/jbc.M106154200 11526113

[62] Courret N Darche S Sonigo P Milon G Buzoni-Gâtel D Tardieux I. CD11c- and CD11b-expressing mouse leukocytes transport single *Toxoplasma gondii* tachyzoites to the brain. Blood. (2006) 107:309–16. 10.1182/blood-2005-02-0666 16051744 PMC1895351

[63] Huynh M-H Carruthers VB. Toxoplasma MIC2 is a major determinant of invasion and virulence. PLoS Pathogens. (2006) 2:e84. 10.1371/journal.ppat.0020084 16933991 PMC1550269

[64] Lavine MD Arrizabalaga G Exit from host cells by the pathogenic parasite *Toxoplasma gondii* does not require motility. Eukaryot Cell. (2008) 7:131–40. 10.1128/EC.00301-07 17993573 PMC2224157

[65] Unno A Suzuki K Xuan X Nishikawa Y Kitoh K Takashima Y. Dissemination of extracellular and intracellular *Toxoplasma gondii* tachyzoites in the blood flow. Parasitol Int. (2008) 57:515–18. 10.1016/j.parint.2008.06.004 18652914

[66] Goldszmid RS Sher A. Processing and presentation of antigens derived from intracellular protozoan parasites. Curr Opin Immunol. (2010) 22:118–23. 10.1016/j.coi.2010.01.017 20153156 PMC2871680

[67] Carruthers VB Håkansson S Giddings OK Sibley LD. *Toxoplasma gondii* uses sulfated proteoglycans for substrate and host cell attachment. Infect Immun. (2000) 68:4005–11. 10.1128/iai.68.7.4005-4011.2000 10858215 PMC101681

[68] Ortega-Barria E Boothroyd JC. A toxoplasma lectin-like activity specific for sulfated polysaccharides is involved in host cell infection. J Biol Chem. (1999) 274:1267–76. 10.1074/jbc.274.3.1267 9880495

[69] Nzoumbou-Boko R Muylder GD Semballa S Lecordier L Dauchy F-A Gobert AP *Trypanosoma musculi* infection in mice critically relies on mannose receptor-mediated arginase induction by a *Tb* KHC1 kinesin H chain homolog. J Immunol. 199:1762–71. 10.4049/jimmunol.1700179 28739879

[70] Bliss SK Butcher BA Denkers EY. Rapid recruitment of neutrophils containing prestored IL-12 during microbial infection. J Immunol. (2000) 165:4515–21. 10.4049/jimmunol.165.8.4515 11035091

[71] Del Rio L Bennouna S Salinas J Denkers EY. CXCR2 deficiency confers impaired neutrophil recruitment and increased susceptibility during *Toxoplasma gondii* infection. J Immunol. (2001) 167:6503–9. 10.4049/jimmunol.167.11.6503 11714818

[72] Khan IA Murphy PM Casciotti L Schwartzman JD Collins J Gao JL. Mice lacking the chemokine receptor CCR1 show increased susceptibility to *Toxoplasma gondii* infection. J Immunol. (2001) 166:1930–37. 10.4049/jimmunol.166.3.1930 11160241

[73] Khan IA Thomas SY Moretto MM Lee FS Islam SA Combe C. CCR5 is essential for NK cell trafficking and host survival following *Toxoplasma gondii* infection. PLoS Pathogens. (2006) 2:e49. 10.1371/journal.ppat.0020049 16789839 PMC1475660

[74] Dunay IR Damatta RA Fux B Presti R Greco S Colonna M Gr1(+) inflammatory monocytes are required for mucosal resistance to the pathogen Toxoplasma gondii. Immunity. (2008) 29:306–17. 10.1016/j.immuni.2008.05.019 18691912 PMC2605393

[75] Eckert C Klein N Kornek M Lukacs-Kornek V. The complex myeloid network of the liver with diverse functional capacity at steady state and in inflammation. Front Immunol. (2015) 6:179. 10.3389/fimmu.2015.00179 25941527 PMC4403526

[76] Zhao Y Marple AH Ferguson DJP Bzik DJ Yap GS. Avirulent strains of *Toxoplasma gondii* infect macrophages by active invasion from the phagosome. Proc Natl Acad Sci USA. (2014) 111:6437–42. 10.1073/pnas.1316841111 24733931 PMC4035997

[77] Groot F Geijtenbeek TB Sanders RW Baldwin CE Sanchez-Hernandez M Floris R. Lactoferrin prevents dendritic cell-mediated human immunodeficiency virus type 1 transmission by blocking the DC-SIGN–Gp120 Interaction. J Virol. (2005) 79:3009–15. 10.1128/jvi.79.5.3009-3015.2005 15709021 PMC548463

[78] Ayechu-Muruzabal V van Stigt AH Mank M Willemsen LEM Stahl B Garssen J. Diversity of human milk oligosaccharides and effects on early life immune development. Front Pediatr. (2018) 6:1–9. 10.3389/fped.2018.00239 30250836 PMC6140589

