# Supplementary material for: CD209 C-Type Lectins Promote Host Invasion, Dissemination, and Infection of Toxoplasma gondii
Source: Front Immunol. 2020 Apr 23;11:656. doi: 10.3389/fimmu.2020.00656 (PMC7190871; doi:10.3389/fimmu.2020.00656)
Supplement: Supplementary file 1 [file Table_1.docx]

**SUPPLEMENTARY MATERIAL**

**RESULTS**


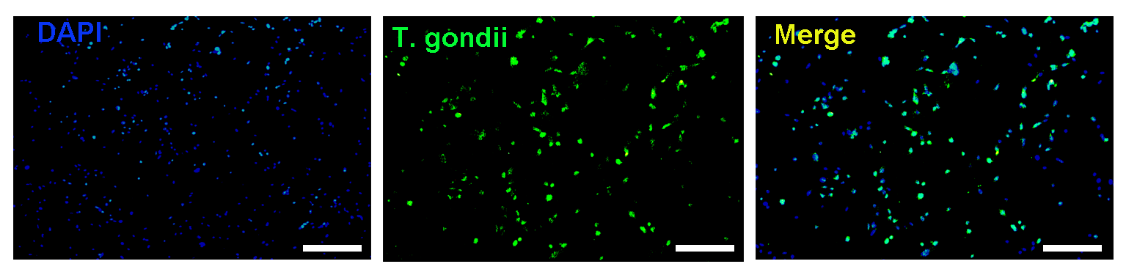


Figure S1: CD09 may be involved in the invasion of *T. gondii* into peritoneal macrophages. A: Cells were fixed and stained for expression of nucleus using DAPI (4’,6-diamidino-2-phenylindole; blue); and B: the GFP-labeled-*T. gondii* (green) within adherent cells (peritoneal macrophages); C:Merged cells with DAPI counterstaining showing fluorescing GFP-labeled *T. gondii*.


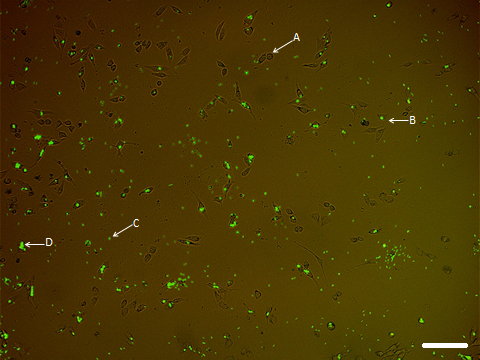


Figure S2: *T. gondii* invasion of CHO-hDC-SIGN. Cells were visualized using white light and the GFP-labeled *T. gondii* were visualized using fluorescent light and the 2 views merged as shown in the figure to illustrate GFP labeled intracellular parasites within adherent cells (CHO vs CHO-hDC-SIGN) and extracellular parasites after 8 hours of co-culture. (A: Adherent cells on the wall of the plate; B: *T. gondii* infected cell; C: Extracellular parasite; D: A cluster of parasites infecting a cell); Scale bar is approximately 10µm).


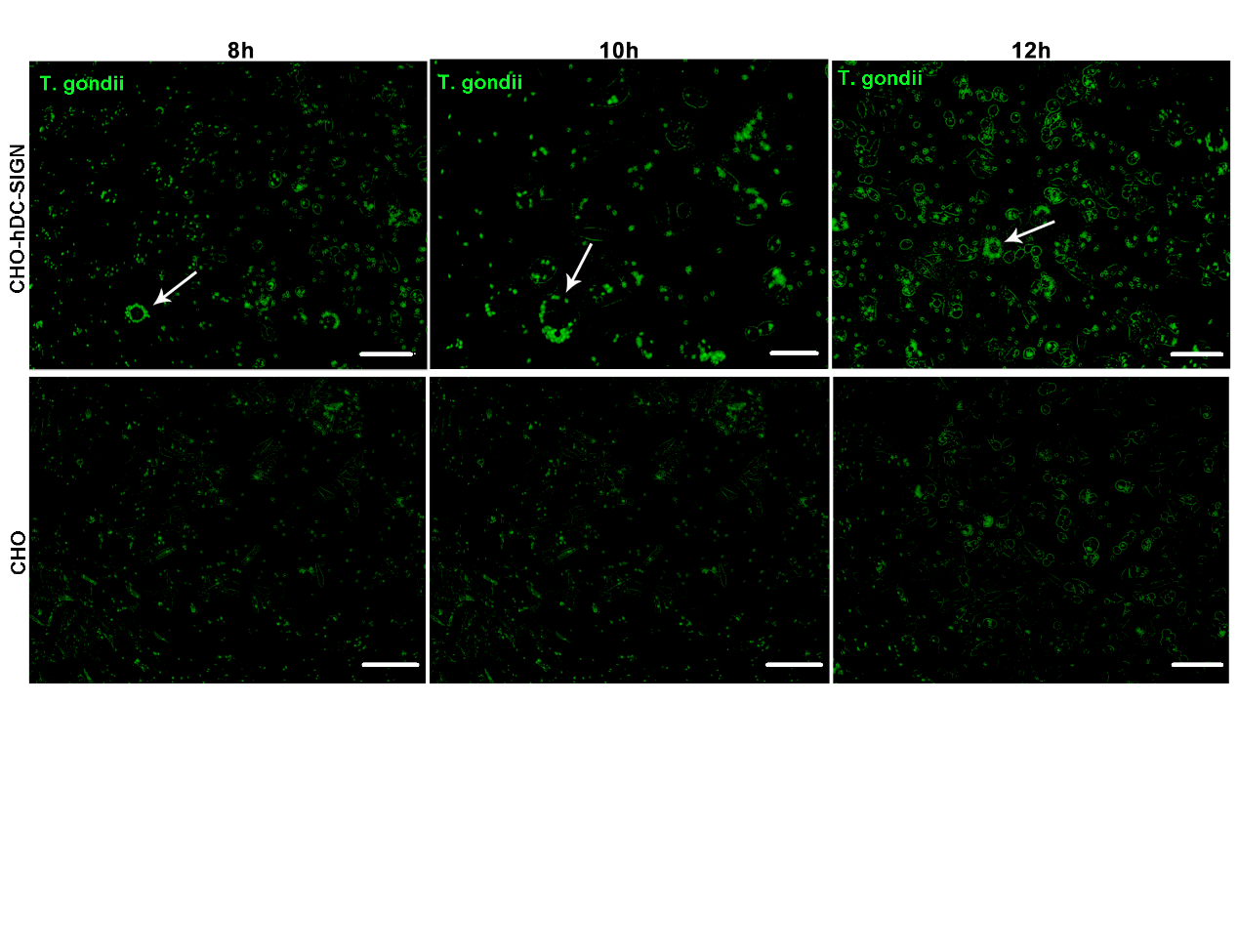


Figure S3: CD09 may be involved in the invasion of *T. gondii* into CHO-hDC-SIGN. Increased *T. gondii* adherence to the cell membrane of CHO-hDC-SIGN in clusters and formation of spherical shapes around the cells. Most of the parasites appear to be located on the cell membrane and not the cytoplasm and also fluoresce more than in CHO cells. Data represent at least three independent experiments with similar results.
